# Measurement & assessment of pain reduction six months following combined scarf akin’s osteotomies +/- 2/3 toe correction for hallux valgus

**DOI:** 10.1186/1757-1146-7-S2-A6

**Published:** 2014-11-14

**Authors:** Irvine Nake, Derek Santos, Gary Boon, Francis Babi, David Cartwright, Anthony Maher, Lee Murphy, Tosin Adekunle, Martin Murgatroyd, Sally Plant, Jackie Ludlam, Mavis Clark

**Affiliations:** 1 Department of Podiatric Surgery, Doncaster & Bassetlaw Hospitals NHS Foundation Trust, Doncaster, South Yorkshire, UK; 2 Department of Podiatric Medicine, Faculty of Health Sciences, Queen Margaret University, Musselburgh, Scotland, UK; 3 Department of Podiatric Surgery, Rotherham NHS Foundation Trust, Rotherham, UK; 4 Podiatric Surgery Unit, Derbyshire Community Health Services NHS Trust, Buxton, UK; 5 Department of Podiatric Surgery, Nottinghamshire Healthcare NHS Trust, Nottingham, UK; 6 Department of Podiatric Surgery, Norfolk Community Health and Care NHS Trust, Norwich, UK

## Background

Hallux valgus deformity is not a single disorder as the name might imply, but a complex multifactorial deformity of the first ray that is often accompanied by deformity and symptoms of pain even in the lesser toes. Research into foot pain has been limited by the lack of a clear understanding as a whole as to what constitutes foot problems. The aim of this study was to measure the effects of the combined Scarf and Akin’s osteotomy with or without 2/3 toe correction for Hallux valgus deformity at 6 months period. Outcome measures used were the pain scale (VAS) and the Manchester-Oxford Foot Questionnaire (MOXFQ).

## Methods

The study was a prospective design and included 30 patients aged 18 to 65+ years with painful bunions plus or minus lesser toe involvement with foot deformity in the study who went on to be treated by the above mentioned surgical procedure with normal heel postoperative weightbearing in a stiff soled surgical shoe during a 6 months period. Mean age of patients at the time of surgery was 59 years, 25 patients were female and 4 were male. History and physical pre-operative assessments (clinical and radiographic) including outcome measures (VAS & MOXFQ) results were performed both at baseline and at 6 months. Post-operative management of the patients was as per normal guidelines set by the department of Podiatric Surgery following a reconstructive bunion surgery.

## Results

The patient related outcome measures, VAS and the MOXFQ questionnaire for the cohort clearly showed statistical significances following foot surgery. The VAS pain scale domain, the median based on the post-surgical scores, was reduced to 0 (IQR 0) with a score change of -6 (IQR 3) (P<0.001). The MOXFQ pain domain, the median based on the post-surgical scores, was reduced to 5 (IQR 0) with a score change of -55 (IQR 27) (P<0.001). The MOXFQ walking and standing domain, the median based on the post-surgical scores was reduced to 0 (IQR 15) with a score change of -50 (IQR 28) (P<0.001). The MOXFQ social interaction domain, the median based on the post-surgical scores, was reduced to 0 (IQR 7) with a score change of -50 (IQR 25) (P<0.001). No post-operative complications were observed, only one patient was lost to post op follow up and her data was discarded.

## Conclusion

A combined Scarf Akin osteotomy with or without 2/3 toe is an effective procedure for the correction of symptomatic Hallux valgus foot deformity at 6 months. It permits early weight bearing of the treated extremity and it requires exact pre-operative planning and strict adherence to the operative technique if pain is to be effectively eliminated and the HRQOL restored with above satisfactory results.

